# A cluster of children with facial nerve palsy in a high prevalence area for COVID-19

**DOI:** 10.1186/s12887-021-02831-9

**Published:** 2021-10-25

**Authors:** David Barron, Owen Richards, Fleur Archer, Mohamed Abdelrazek, Rajesh Ranjan, Omotakin Omolokun

**Affiliations:** 1 School of Medicine, Cardiff University, Cardiff, CF14 4XW UK; 2 Cwm Taf Morgannwg University Health Board, Paediatrics department, Royal Glamorgan Hospital, Ynysmaerdy, UK

## Abstract

**Background:**

COVID-19 is a disease of varying presentation and neurological sequelae of the disease are being studied. Following a cluster of paediatric facial nerve palsy (FNP) cases in an area of South Wales with a high prevalence of COVID-19, we conducted an opportunistic study to determine whether there has been an increase of incidence of FNP and if there is an association between the FNP and COVID-19 in children.

**Methods:**

We performed a retrospective review of the incidence of FNP between 2015 and 2020 across two hospitals within the health board. The incidence was compared with that in 2020 including a cluster of six children in 14 weeks, presenting to Royal Glamorgan Hospital between June and October.

**Results:**

There were 48 cases of children with FNP across both hospital within the study years. Seven (7) cases in 2020. The incidence was not statistically different in comparison to other years.

Five out of six of these children in 2020 had antibody testing for COVID-19. All serology testing (100%) returned negative for SARS-CoV- 2 antibodies.

**Conclusions:**

In high prevalence area for COVID-19, cases of children with FNP have not shown a commensurate increase. we have found no causal link between COVID-19 and FNP in children. While this is a small study, larger cohort studies are needed to support this finding.

As new strains of COVID-19 are being reported in UK, South Africa and Brazil, physicians need to continue to be vigilant for consistent pattern of signs and symptoms, especially in children.

## Background

To date, COVID-19 infections have passed 84 million cases, with over 1.8 million deaths reported worldwide [1]. While most patients experience mild symptoms or are asymptomatic [2], there is emerging research of differing sequelae of the viral illness. Neurological syndromes that have been associated with COVID-19 [3] include anosmia, ageusia, Guillain Barré syndrome, encephalopathy [4], facial nerve palsies (FNPs) [5, 6] and “long COVID” [7].^.^

As a group of medical students placed in the Paediatric Assessment Unit in the Royal Glamorgan Hospital (RGH), Wales, with the highest prevalence of COVID-19 in South Wales at the time (including remarkably high rates of intra-hospital transmission), we were surprised at the number of children presenting with FNP. According to the paediatric team, this was not a common occurrence. Thus, we reviewed these cases to see if there had been a significant increase in the incidence of FNP in 2020, in comparison to previous years, and whether these cases were related to the COVID-19 pandemic as a neurological sequela of the infection.

## Methods

This was an opportunistic study conducted on the Paediatric ward of the RGH in Wales. It took place between June and October 2020, wherein we collected data from six children with FNP. Each patient was diagnosed, treated and followed up using the same systematic structure, at different points in time. All attendees were required to complete a COVID-19 health-check questionnaire, prior to assessment on the same day of referral. Children with high scores were offered COVID-19 testing.

A clinical diagnosis of FNP was made in all six children, during a 14-week period. The severity of FNP was documented using the House-Brackmann score. Blood pressure checks and a full blood count were done as part of routine care, before commencement of a weeklong course of steroid treatment, following evidence based guidelines [8].

All patients were followed up at one and 4 weeks to check for resolution of facial weakness, and after gaining consent, antibody testing for COVID-19 was performed on them upon recall.

The incidence of FNP in 2020 was compared to previous years’ statistics, using statistical package SPSS26. An electronic search was performed for children aged 0–16 years with a clinical coding discharge diagnosis of ‘Facial Palsy’, ‘Bell’s Palsy’, and ‘Idiopathic facial nerve palsy’, between the years 2015 to 2020, across two hospitals in Wales – RGH and Prince Charles Hospital (PCH). Children with traumatic birth injuries causing palsy, and children with a secondary diagnosis, such as cerebral palsy, Chiari malformation and other syndromes were excluded from the study.

## Results

There were 48 cases of FNP across both hospitals from the year 2015 to 2020: 30 in RGH and 18 in PCH. The demographics of the patients can be seen in Table 1. Of the six cases in RGH, between June and October 2020, a chi-squared test demonstrated that the change in the incidence of FNP across the years is non-significant (*p* value = 0.263).

**Table 1:**
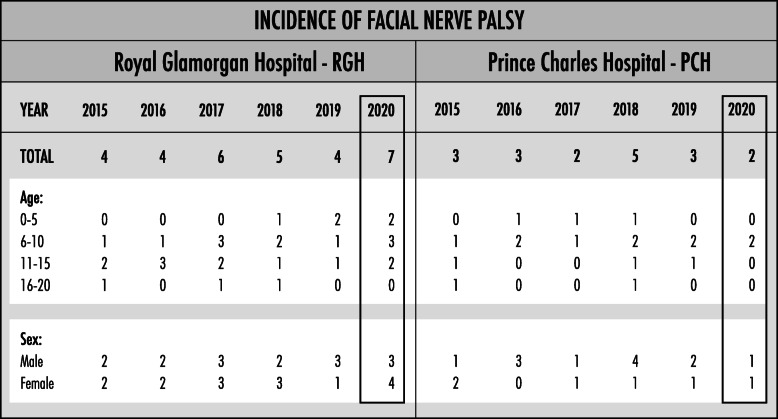
Demographics of Children with Facial Nerve Palsy. Patients Across Two Hospitals in Cwm Taf Morgannwg University Health Board

In the year 2020, two of the six children underwent COVID-19 testing during the episode of FNP, with negative results. One of the six children tested positive 3 months after the initial presentation with FNP and is unlikely to be related.

Five out of six children underwent antibody testing for COVID-19, performed at least 2 weeks after initial presentation with FNP. All serology testing (100%) returned negative for SARS-CoV-2 antibodies.

## Discussion

This retrospective cohort study conducted across two hospitals found no causal relationship between COVID-19 and FNP in the paediatric population. The incidence of FNP in 2020 was not significantly different to the previous 5 years. Of the patients admitted in 2020 with confirmed FNP, no evidence of COVID-19 infection was detected on admission, nor retrospectively via a SARS-CoV-2 antibody screen.

While COVID-19 is a disease of highly variable presentation, ranging from asymptomatic infection to multisystem organ failure [9], peripheral neurological disturbance is widely observed in adult patients with anosmia and hypogeusia recognised as common symptoms [10, 11]. Polyneuropathies, including Guillain-Barré, polyneuritis cranialis and Miller Fisher syndrome, have also been reported as neurological manifestations of COVID-19 in adults [12–14]. Although literature on similar presentations in a paediatric population is scarce, cases of altered taste and smell in the absence of other symptoms have been described in children with confirmed SARS-CoV-2 infection [15, 16]. Additionally, children who become critically unwell after developing a systemic inflammatory response to COVID-19, known as Paediatric Multisystem Inflammatory Syndrome (PIMS-TS) – temporally associated with SARS-CoV-2 – exhibited global proximal muscle weakness and reduced reflexes [17]. These neurological sequelae, albeit rare, are concerning.

To date, only one case of FNP has been reported in a paediatric patient with COVID-19 [18]. Although FNP is commonly idiopathic, infections such as Herpes Simplex Virus-1, Varicella Zoster Virus and Lyme disease are established causes of facial paralysis [19]. As the clinical manifestations of infection with SARS-CoV-2 are still being elucidated, it is reasonable to surmise its possible involvement with FNP. Indeed, in adults, several case reports have described individual instances of unilateral facial palsy in the onset of COVID-19 [20–22]. In a case series conducted in Brazil, Lima et al. [5] described eight patients with COVID-19 who developed FNP during the illness. Similarly, Zammit et al. [23] showed a significantly elevated incidence of FNP in 2020, as compared to 2019, and proposed the COVID-19 pandemic as a reason for the increase. However, of their 30 patients, only 17 (57%) were tested for COVID-19 and only two returned a positive result. In all the studies, proving a definitive correlation was hindered due to a small sample size.

Whilst a notable cluster of paediatric patients presenting with unilateral facial nerve paralysis was observed from June to October 2020, this study found no association with the ongoing pandemic. Not only were none of the patients positive for COVID-19 on admission, subsequent testing for SARS-CoV-2 antibodies was also negative. Even though it has been suggested that FNP should be recognised as a presenting sign of COVID-19 in children [18], our data suggests otherwise. Larger scale studies with similar results would most likely rule out an association. Of note is that the 27 children with positive COVID-19 results at the RGH did not show any neurologic symptoms (unpublished data).

The limitation of this study was a small sample size, and some antibody tests were performed within the recommended six-week waiting period, potentially allowing insufficient time for detectable immunoglobulin G SARS-CoV-2 antibodies to be produced.

## Conclusion

Our study has not been able to show any causal link between isolated FNP and COVID-19; however, our results should be interpreted with caution as they provide a reassuring insight into the neurological manifestations of COVID-19 in a paediatric population and suggests that FNP in children has no association with this unpredictable disease. We are reassured by overwhelming evidence that only very rarely do children show severe symptoms of COVID-19, such as seen with PIMS-TS. However, as new variants are reported in the UK with increased transmissibility, clinicians need to continue to be vigilant for clinical patterns of this emerging disease, especially in the paediatric population.

## Data Availability

The datasets generated and/or analysed during the current study are not publicly available due [to protection of personal data and fairness.] but are available from the corresponding author on reasonable request.

## Abbreviations

SARS-CoV-2: Severe acute respiratory syndrome coronavirus 2
PIMS-TS: Paediatric multisystemic inflammatory syndrome temporally associated with SARS-CoV-2
FNP: Facial Nerve palsy
RGH: Royal Glamorgan Hospital
